# Integrative network pharmacology and experimental validation reveal emodin derivatives as potential therapeutics for hepatocellular carcinoma

**DOI:** 10.1186/s43046-025-00306-x

**Published:** 2025-07-21

**Authors:** Wirawan Adikusuma, Firdayani Firdayani, Siska Andrina Kusumastuti, Nuralih Nuralih, Shelvi Listiana, Ayu Masyita, Lalu Muhammad Irham, Siti Hodijah, Suci Zulaikha Hildayani, Eko Mugiyanto

**Affiliations:** 1 Research Center for Computing, Research Organization for Electronics and Informatics, National Research and Innovation Agency, Cibinong, Indonesia; 2 Department of Pharmacy, Universitas Muhammadiyah Mataram, Mataram, Indonesia; 3 Research Center for Vaccine and Drugs, Research Organization for Health, National Research and Innovation Agency, South Tangerang, Indonesia; 4 Research Center for Raw Pharmaceutical and Traditional Medicine, Research Organization for Health, National Research and Innovation Agency, South Tangerang, Indonesia; 5 Faculty of Pharmacy, Universitas Ahmad Dahlan, Yogyakarta, Indonesia; 6 Study Program of Biophysics, Department of Physics, Faculty of Mathematics and Natural Sciences, IPB University, Bogor, Indonesia; 7 Pharmacist Professional Education Program, Faculty of Health Sciences, Universitas Muhammadiyah Pekajangan Pekalongan, Pekalongan, Indonesia

**Keywords:** Hepatocellular carcinoma, Emodin derivatives, Network pharmacology, Cytotoxicity assay, Molecular docking, Molecular dynamics simulation

## Abstract

**Background:**

Hepatocellular carcinoma (HCC) is a major global health concern due to its high prevalence and mortality rate. Although emodin, a natural anthraquinone derivative, has demonstrated in vitro anticancer activity against HCC cells, its specific molecular targets in HCC remain unclear.

**Method:**

This study used an integrated approach combining in silico network pharmacology, molecular docking, molecular dynamics simulations (MDS), and in vitro cytotoxicity assays to evaluate three emodin derivatives: emodin, 3-acetyl emodin (ACE), and 1,3,8-triacetyl emodin (TAEM). Target predictions were performed using the SwissTargetPrediction database, and HCC-related genes were retrieved from cBioPortal. Functional annotations (Gene Ontology and Reactome) identified *EGFR* and *KIT* as key targets. Docking simulations were conducted to assess binding affinities, followed by 100 ns MDS to evaluate stability. Cytotoxic effects on HepG2 cells were also assessed.

**Result:**

TAEM showed the strongest binding affinity to both *EGFR* and *KIT* and demonstrated the highest cytotoxicity against HepG2 cells (IC50 = 0.021 mM). MDS results indicated that the KIT-TAEM complex was the most stable among all tested combinations, supported by RMSD, RMSF, Rg, protein–ligand distance, and MM-GBSA binding energy analyses.

**Conclusion:**

These findings highlight TAEM as a promising therapeutic candidate for HCC. The study demonstrates the value of integrating computational predictions with experimental validation in early-stage drug discovery.

**Supplementary Information:**

The online version contains supplementary material available at 10.1186/s43046-025-00306-x.

## Introduction

Liver cancer is becoming more common worldwide and will affect almost one million people each year by 2025. The main type of liver cancer is hepatocellular carcinoma (HCC), which accounts for 90% of cases [1–3]. About 50% of HCC cases are caused by the hepatitis B virus (HBV) [4]. Treatment with antiviral medication has reduced the risk of HCC caused by another virus, hepatitis C (HCV), but people with liver scarring (cirrhosis) are still at risk [5]. In some countries, the most common cause of HCC is non-alcoholic steatohepatitis (NASH), which is linked to diabetes and metabolic syndrome [6]. Besides, smoking and exposure to certain chemicals can also cause HCC [7]. HCC is the sixth most common cancer in the world and the fourth leading cause of cancer deaths [3]. The highest rates of HCC are in East Asia and Africa, but the number of cases is increasing in other parts of the world, including the United States, where HCC is the fastest-growing cause of cancer-related deaths [8]. Currently, surgery-based approach is the best choice for the treatment of HCC progression, but many people are detected too late drive to not only difficulty to diagnose but also to treat patients. Furthermore, traditional chemotherapeutic treatments have many drawbacks including adverse effects and drug resistance, making them unsatisfactory [9, 10]. Thus, finding new HCC-active compounds or therapeutic techniques with lesser toxicity is vital for prevention and treatment.

**Fig. 1:**
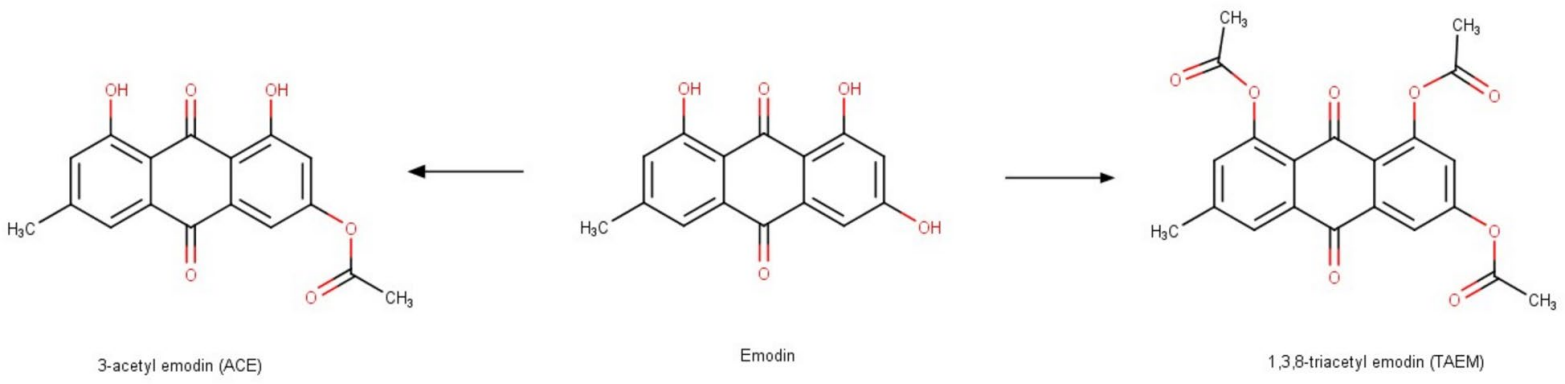
Structural modification of Emodin

Natural products have been used extensively to make new drugs for various diseases like cancer. One of these natural substances is Emodin, a natural anthraquinone derivative. It is found in traditional Chinese medicines like *Rheum palmatum*, *Polygonum cuspidatum*, and *Polygonum multiflorum* [11], which have demonstrated strong anticancer effects in different cancers both in vivo or in vitro [12–15]. In addition, Emodin may play a role in HCC, but its molecular target has not been proven [16], and little research has explored this possibility. In particular, by modifying the structure of Emodin, it could be suggested novel derivatives that are more effective in treating HCC using the specific molecular target. The structural modification was done by replacing the hydroxyl with an acetyl group as shown in Fig. 1.

On the other hand, a network pharmacology-based approach could guide the drug discovery process. It can lead to development of new drugs with enhanced efficacy and fewer side effects. Network pharmacology is a scientific approach that uses complex biological systems and network analysis of multiple drug targets to predict the molecular targets of new chemical entities and disease pathways [17]. Additionally, this method involves molecular docking analysis, which allows for predicting potential intermolecular interactions between a target and compounds [18]. Moreover, molecular dynamics simulation (MDS) has become an essential complementary tool, providing deeper insights into the stability, flexibility, and conformational changes of protein–ligand complexes over time [19]. These in silico methods collectively contribute to reducing the time and cost associated with preclinical research. In this study, we integrated network pharmacology, bioinformatics, molecular docking, and MDS to elucidate the potential molecular mechanisms of emodin and its derivatives against HCC.

## Material and methods

### Acetylation of emodin

Approximately 1 mmol of Emodin was dissolved in 6 mmol of pyridine. Ten mmol of acetic anhydride was added and the mixture was reacted at room temperature for 4 h to obtain 3-acetyl emodin (ACE) or 24 h to obtain 1,3,8-tri acetyl emodin (TAEM). Ice and concentrated HCl were added to the solution to stop the reaction, and a precipitate formed. The precipitate formed was washed with dilute HCl to remove pyridine residue. The crystals formed were recrystallized using a hot ethyl acetate solvent. Qualitative analysis of the synthesis results was carried out using the TLC and HPLC methods by observing the presence of single spots or peaks that differed from the starting material (Emodin). The purified synthesis products confirmed their structure using LC–MS/MS, FTIR, and NMR [20, 21].

### Identification of molecular target

The Swiss Target prediction system, which predicts pharmacological targets based on similarities, was used to screen Emodin, ACE, and TAEM. The compounds'canonical SMILES was uploaded to the Swiss Target Prediction database (http://www.swisstargetprediction.ch/; accessed on February 14, 2023) to predict protein targets of compounds [22].

### Identification of HCC target genes

HCC target genes with somatic mutations were identified using the cBioPortal database (https://www.cbioportal.org/; accessed February 14, 2023), known as HCC-associated genes. The free cBioPortal contains cancer genomics data sets. Around 5,000 tumor samples from 20 cancer studies are available [23]. Cancer genomic data from cBioPortal could be used to gain biological insights and therapeutic applications.

### Prioritizing target genes by using functional annotations

Four functional annotations: Biological process (BP), Cellular component (CC), Molecular functions (MF), and Reactome were used to discover potential target genes for HCC. A higher annotation score means that genes have a bigger effect on how HCC develops. This study looked at functional annotations like BP, CC, MF, and Reactome using the WebGestalt 2019 functional enrichment analysis tool (http://www.webgestalt.org/; accessed on February 14, 2023) [24]. The first three criteria (BP, CC, and MF) are all part of gene ontology (GO) and were labeled to help figure out how proteins interact with each other. Reactome was done to find out which gene affects the molecular pathway.

### In silico* molecular docking analysis*

The molecular docking between two protein targets, *EGFR* (PDB ID: 1M17) and *KIT* (PDB ID: 4U0I), with Emodin, ACE, and TAEM as ligands has been performed using Molegro Virtual Docker program. The ligand structures have been drawn in 2D, then converted to 3D and the most stable conformation is determined. The docking procedure is carried out in two steps, the first is cavity detection, followed by docking simulation, which excludes all water molecules. Next, docking analysis was performed to determine the minimum binding energy and intermolecular interactions occurring in the protein–ligand complex, such as the presence of hydrogen bonds. The Method validation was carried out by conducting the molecular docking simulation for native ligand. The docking method is valid if the RMSD value is less than 2 Å.

### MTT assay validation

The Human hepatocarcinoma (HepG2) cell lines were obtained from the Development of Agroindustry and Biomedicine Laboratory (LAPTIAB BRIN, Serpong Indonesia). Cells were cultured in Dulbecco's modified Eagle's medium (DMEM, Gibco, New York, USA) supplemented with 1% penicillin/streptomycin (PS, 100 units of penicillin/mL and 100 µg streptomycin/mL, Gibco, New York, USA) and 10% Fetal Bovine Serum (FBS, Gibco, New York, USA) at 37 °C in a humidified 5% CO_2_ atmosphere. The cytotoxicity assay of HepG2 cells was conducted using MTT colorimetric assay. 1 × 10^5^ cells/well were seeded in a 96-well plate and incubated for 24 h at 37 °C in a humidified 5% CO_2_ atmosphere. Emodin and its derivatives (ACE and TAEM) were added to a 96-well plate in a range of concentrations (0.025–0.8 mM) in FBS-supplemented DMEM and incubated for 24 h. Subsequently, the culture medium was removed and replaced with fresh culture medium containing 0.5 mg/mL MTT. After a 4-h incubation, SDS 10% was added to dissolve the MTT formazan. After incubation overnight at room temperature and in dark conditions, the absorbance value was measured at 570 nm using an ELISA plate reader.

### Molecular dynamics simulations

In this study, the protein–ligand complex with the best binding affinity was subjected to MDS using AMBER24 software. The ligand structure was modeled with General AMBER Force Field (GAFF) [25], while the protein structure was modeled with Amber 14SB force field [26]. The protein structure was solvated on the H + + server [27] in a TIP3P water box [28] with a buffer distance of 10 Å and 0.15 M NaCl solution containing Na⁺ and Cl⁻ to neutralize the charge. The solvated protein–ligand complex underwent 1000 energy minimization steps and was continued with gradual heating from 100 K to 310.15 K for 500 ps. The system was then relaxed with the NPT ensemble and subjected to gradual positional restraints of 100 kcal/mol/Å2, 10 kcal/mol/Å2, 1 kcal/mol/Å2, 0.1 kcal/mol/Å2 until the relaxation was carried out without restraint on the system, each for 500 ps.

The Langevin thermostat was used to keep the system temperature constant at 310.15 K, with a collision frequency of 1 ps⁻1. Meanwhile, the pressure setting was carried out using the Monte Carlo barostat. In this simulation, a time step of 1 fs was used and the SHAKE algorithm was used to lock all bonds involving hydrogen atoms. The Particle Mesh Ewald (PME) method was used to calculate long-range electrostatic interactions in the periodic system with a cutoff distance of 8 Å [29]. Next, the production stage for each protein–ligand complex was carried out for 100 ns in the NPT ensemble, and the trajectory results were analyzed using cpptraj [30].

### Binding free energy calculation (MMGBSA)

The AMBER24 software was used with the MM-GBSA method [31] to analyze the binding free energy of the protein–ligand complex. The total binding free energy of the protein–ligand complex was calculated as follows:

1 $$\Delta G_{binding} = G_{complex} - \left( {G_{protein} + G_{ligand} } \right)$$

where $G_{complex}$, $G_{protein}$, and $G_{ligand}$ are the binding free energies of the complex, target protein, and ligand, respectively. The free energy is formulated as:

2 $$G = E_{GB} + E_{SA} + E_{ele} + E_{vdw}$$

where $E_{GB}$, $E_{SA}$, and $E_{vdw}$ are the generalized Born solvation energy, surface area energy, electrostatic energy, and van der Waals energy, respectively.

## Results

### Acetylation of emodin

The structure of the product of emodin acetylation has been confirmed as reported in previous studies [20, 21]. ACE and TAEM were yellow-orange solids that were soluble in hot ethyl acetate and acetone, less soluble in hexane and chloroform, and insoluble in water.

### Identifying HCC drug targets

**Fig. 2:**
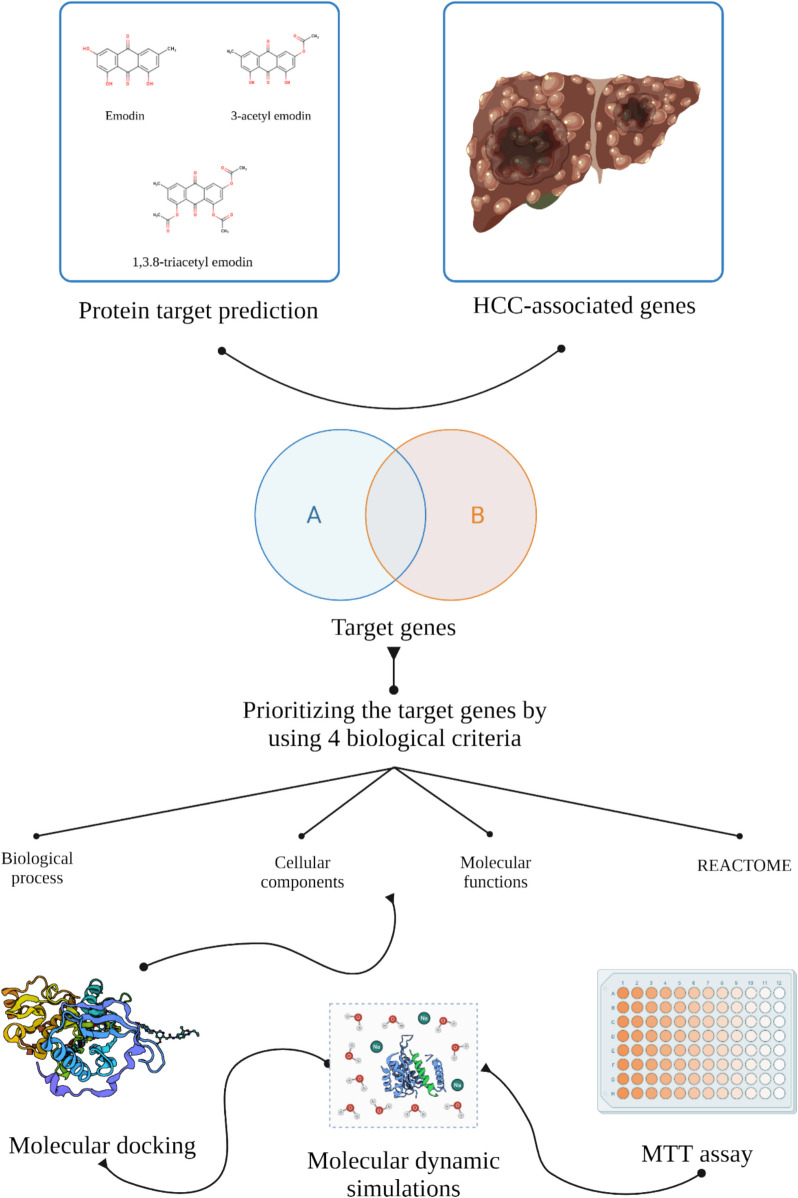
The study workflow to identify potential target emodin in HCC. BioRender.com made this figure under the agreement"ZJ286 KEXD2

**Fig. 3:**
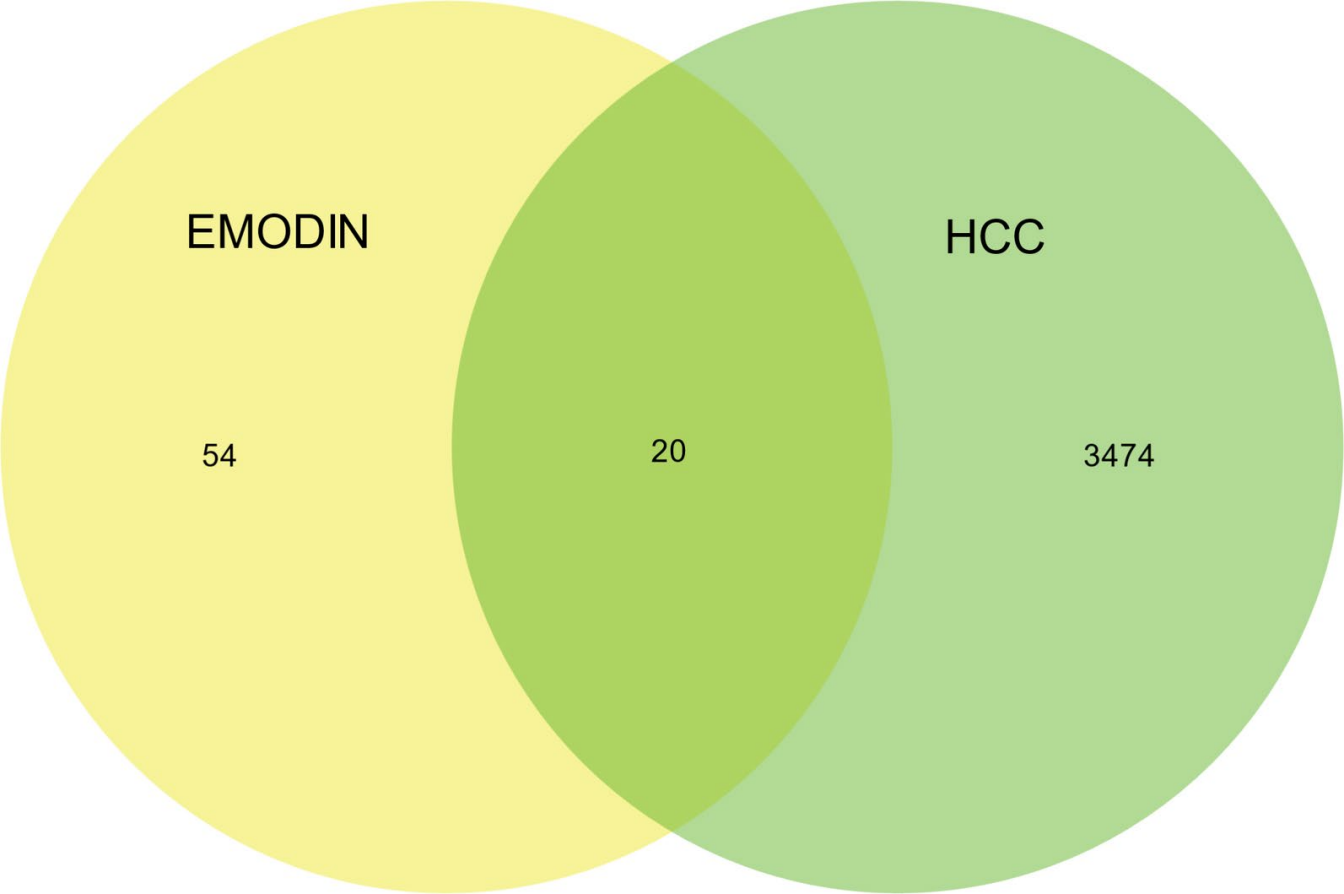
Venn diagram demonstrating the intersection of compound targets (yellow) and HCC targets (green)

The outline of the experimental steps is depicted in Fig. 2. This study used the cBioPortal database to look for targets involved in HCC. We found information from eight different studies about a type of liver cancer called HCC. In total, 1,058 patients participated in these studies. The studies identified a total of 17,653 genes that were mutated in HCC, but we focused on 3494 genes most strongly linked to HCC (Supplementary Table 1). We only looked at genes that were mutated in at least 1% of the patients. The Swiss Target Prediction was used to find out what each of Emodin's targets was. There were 56 predicted targets for Emodin, 27 predicted targets for ACE, and 1 predicted target for TAEM (Supplementary Table 2). The 20 targets of Emodin and HCC that overlapped were shown in a Venn diagram (Fig. 3) and written down (Supplementary Table 3).

### Prediction of emodin and HCC-related targets

This study was performed on 20 specific target genes involved in the relationship between Emodin and HCC. To identify the most important genes, we used four different methods known as functional annotations: BP, CC, MF, and Reactome. Based on these functional annotations, we assigned scores to each gene, with the following results: 10 genes were prioritized by BP, 5 genes by CC, 14 genes by MF, and 3 genes by Reactome. We then identified the three genes with the highest scores: *EGFR* and *KIT* (Table 1).

**Table 1:**
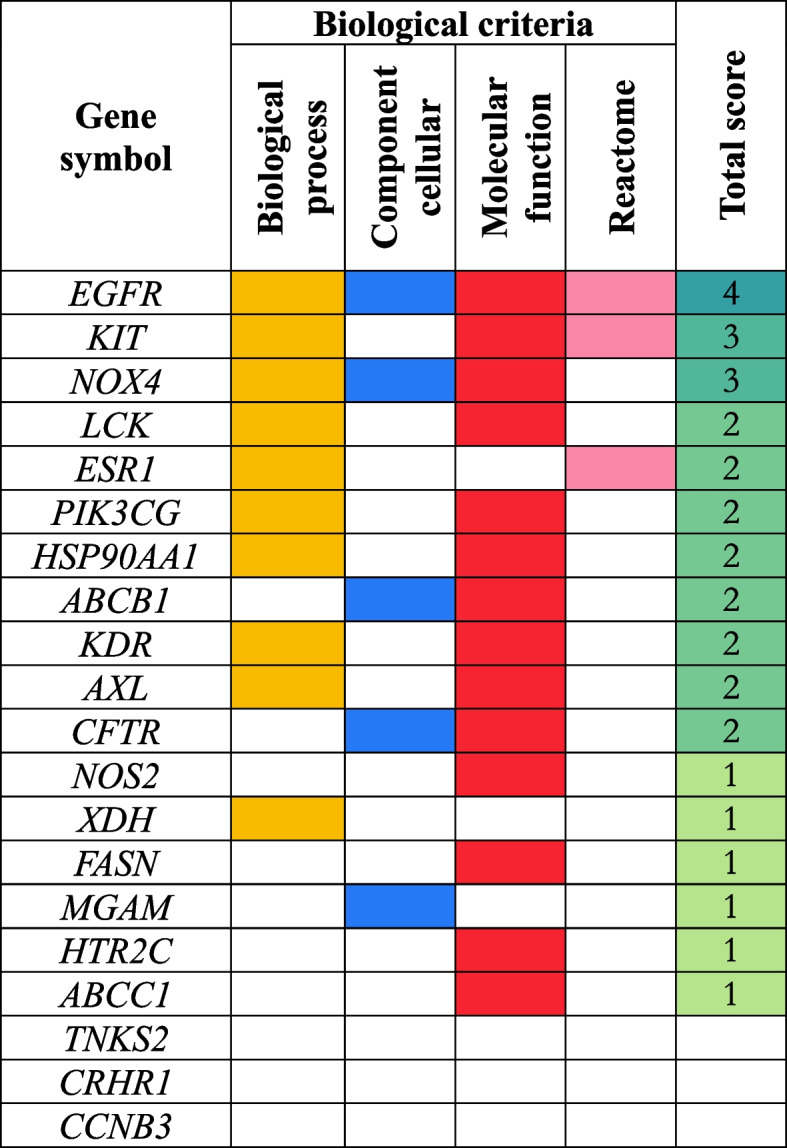
Functional annotations to prioritize compound targets in HCC

**Fig. 4:**
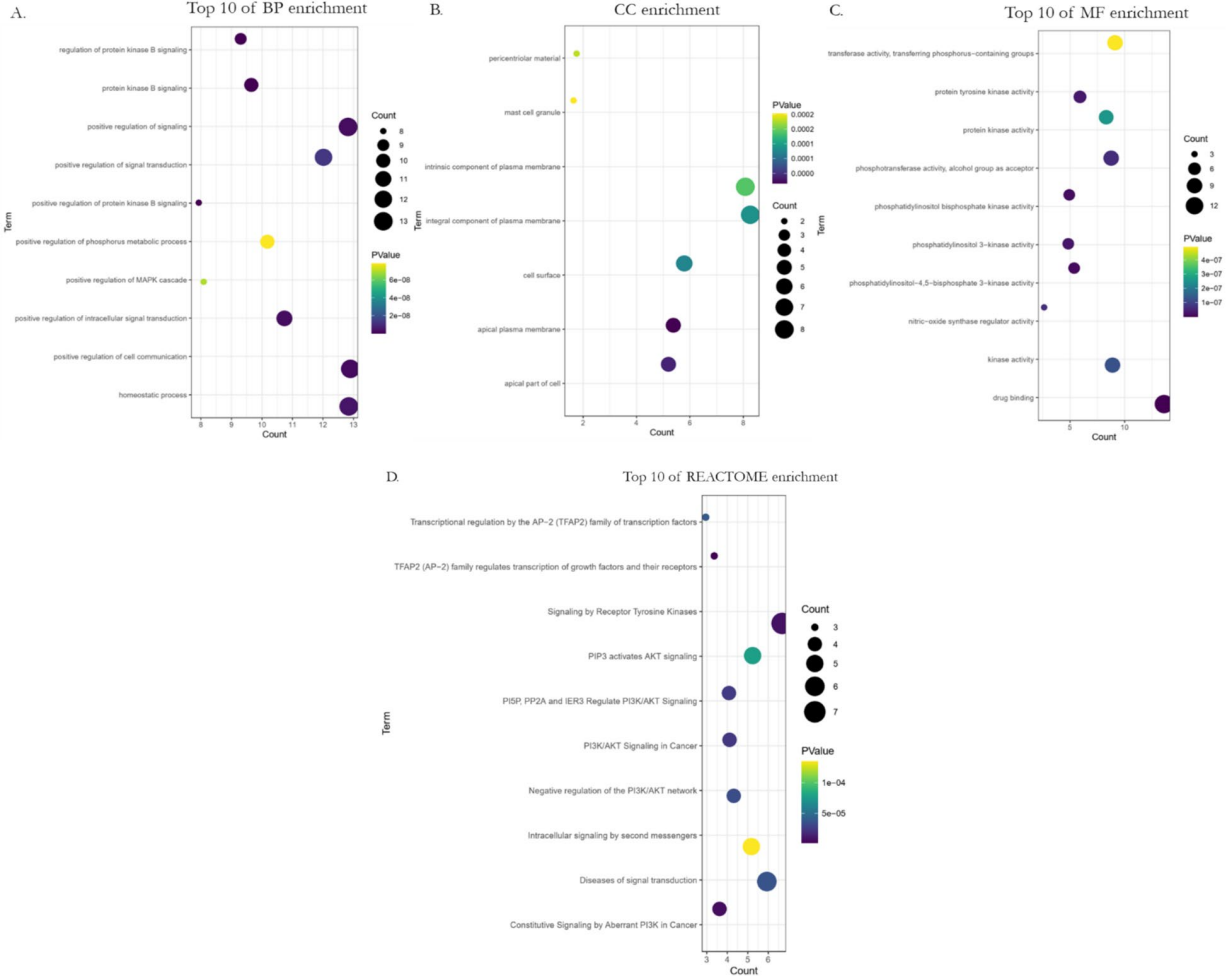
Top 10 significantly enriched analyses for four functional annotations. **A** Biological process; **B** Cellular component; **C** Molecular function; and **D** Reactome

Further analysis of the results showed that these top three genes are strongly associated with cancer development. The results showed that the most significant finding related to the BP method was the involvement of protein kinase B signaling. The CC method showed that the genes were important in a specific part of the cell called the apical plasma membrane. The MF method suggested that the genes were important in drug binding. Lastly, the Reactome method revealed that the genes were involved in a pathway that regulates the growth of cancer cells, particularly the TFAP2 (AP-2) family which controls the transcription of growth factors and their receptors. The top ten functional enrichment analyses are based on the four methods, as shown in Fig. 4A-D. The complete results of the functional enrichment analysis are included in Supplementary Table 4.

### Molecular docking verification

**Fig. 5:**
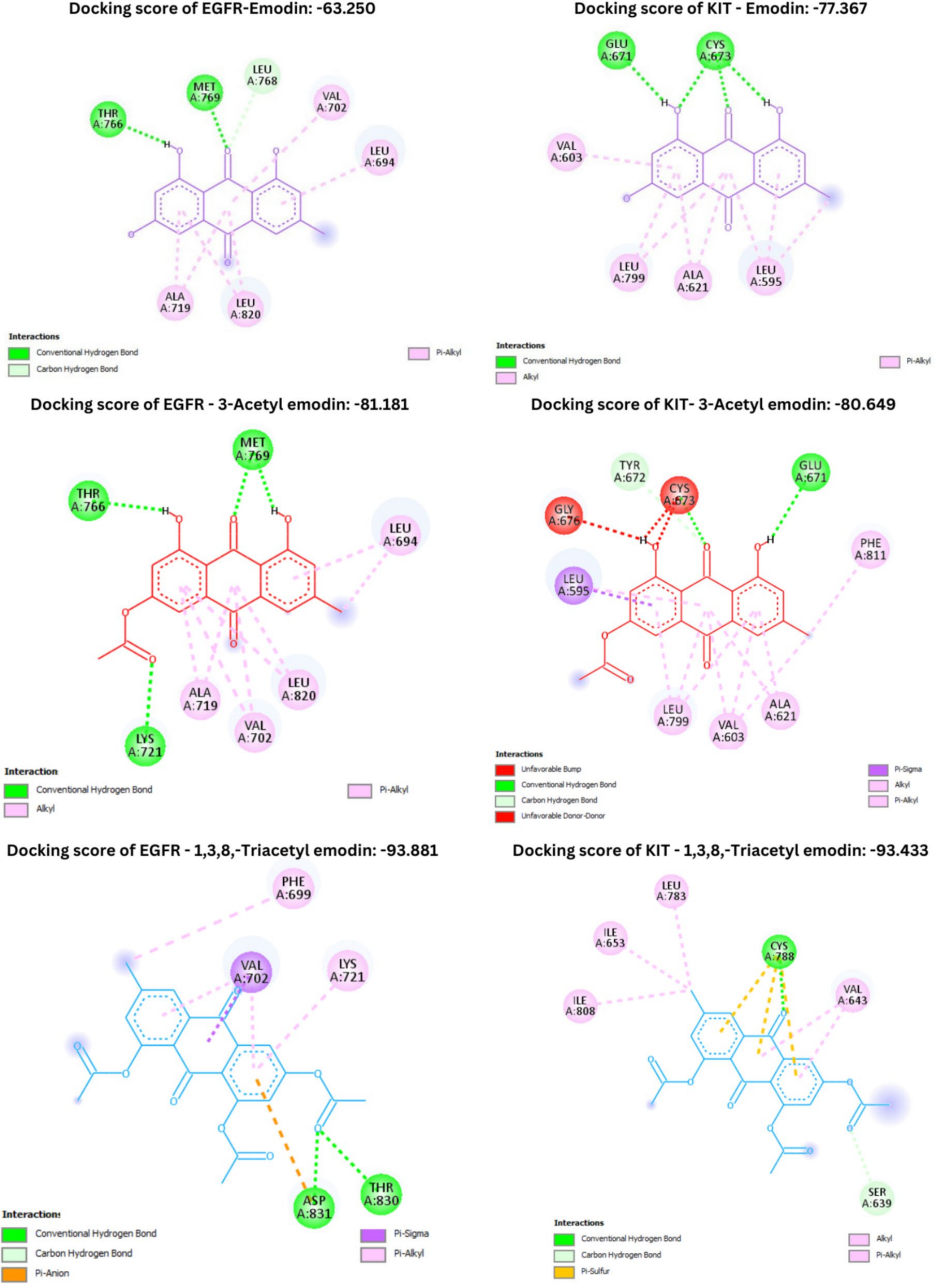
Docking profiles of Emodin, 3-acetyl Emodin, and 1,3,8-tri acetyl emodin with top 2 targets. **A** Emodin with target *EGFR*; **B** Emodin with target *KIT*; **C** 3-acetyl Emodin with target *EGFR*; **D** 3-acetyl Emodin with target *KIT*; **E** 1,3,8-tri acetyl emodin with target *EGFR*; and **F** 1,3,8-tri acetyl emodin with target *KIT*

Molecular docking simulations were carried out to investigate possible interactions between the potential targets of *EGFR* and *KIT* with Emodin, ACE, and TAEM, respectively. Docking to *EGFR* (PDB ID 1M17) was carried out at x = 21.58; y = 0.40; z = 52.48 with a radius of 15 Å [32]. The docking position for *KIT* (PDB ID: 4U0I) is x = 35.57; y = 9.01; z = 46.56 with a radius of 15 Å validated by redocking the native ligand and showing an RMSD of 0.37. The docking score and contacted residues of all the protein–ligand complexes are presented in Fig. 5.

From the docking results, we found that TAEM showed the highest docking score against *EGFR* compared to other compounds. TAEM interact with THR830 and ASP831 by hydrogen bond. Furthermore, the aromatic ring system of this compound makes pi-alkyl interaction with PHE699 and LYS721. Similarly, we also figured out that TAEM showed the highest binding affinity against *KIT*, followed by ACE and emodin with docking scores are −93.881, −81.181, and −63.250, respectively. The binding mode for TAEM to *KIT* was attributed to H-bond interaction with CYS788, while amino acid residues VAL643, ILE653, LEU783, and ILE808 make pi-alkyl interaction.

### Cytotoxic activity assay

**Fig. 6:**
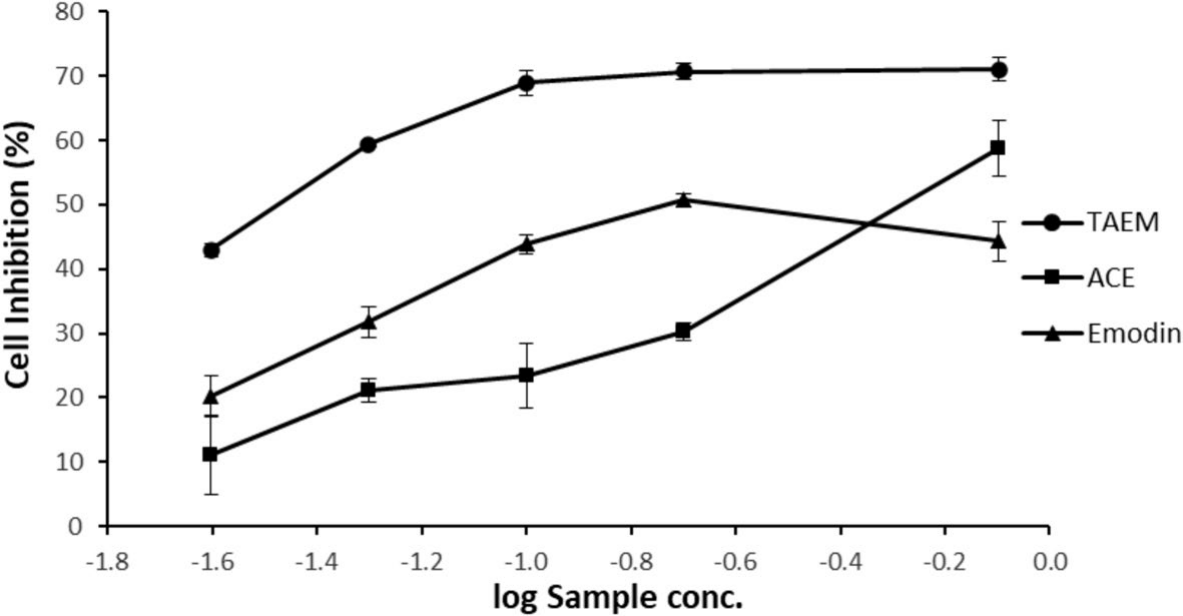
Emodin and its derivatives (TAEM and ACE) inhibited the proliferation of HepG2 cells. HepG2 cells were treated with various concentrations (0.025–0.8 mM) of Emodin, TAEM, and ACE for 24 h. Cell viability was determined using an MTT assay and purple colors from crystal formazan were quantified using an ELISA reader at a wavelength of 570 nm. Data are expressed as the means ± SD. All experiments were done in triplicates

Emodin, ACE, and TAEM were investigated for their inhibitory effects on the proliferation of HepG2 cells. After 24 h of incubation, cytotoxicity assays showed that Emodin, ACE, and TAEM significantly inhibited the growth of HepG2 cells. The dose–response study demonstrated that Emodin inhibited cell growth in a dose-dependent manner up to 0.2 mM, while ACE and TAEM also exhibited growth inhibition in HepG2 up to 0.8 mM (Fig. 6). The IC50 values of Emodin, ACE, and TAEM were 0.603 mM, 0.574 mM, and 0.021 mM, respectively. Furthermore, the cytotoxicity of TAEM against HepG2 cells was found to be higher than that of Emodin and ACE (Fig. 6), which suggests that TAEM may be a potential target for the treatment of HCC.

### Molecular dynamics simulation results

**Fig. 7:**
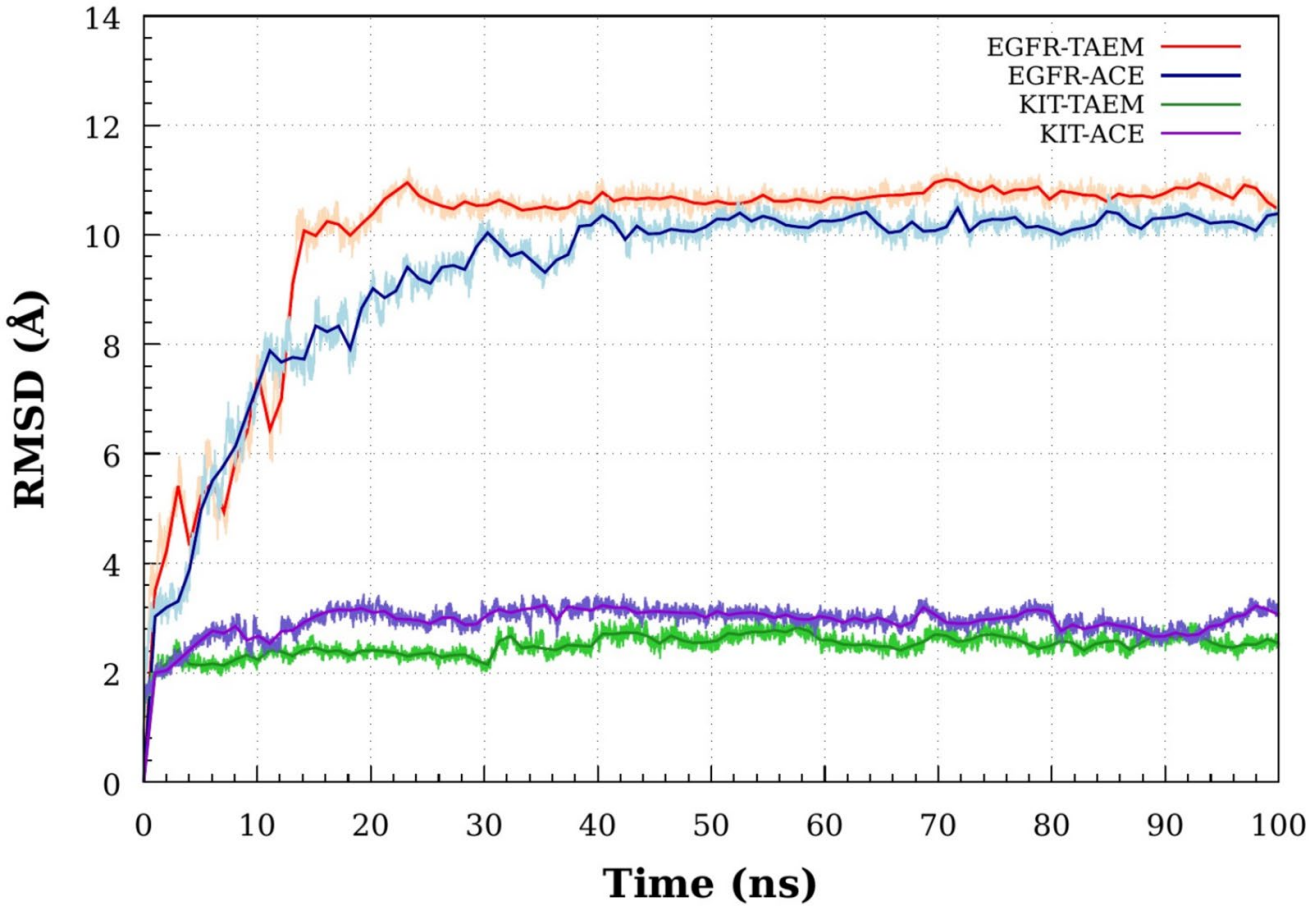
RMSD plot of *EGFR*-ligand and *KIT*-ligand complexes

**Fig. 8:**
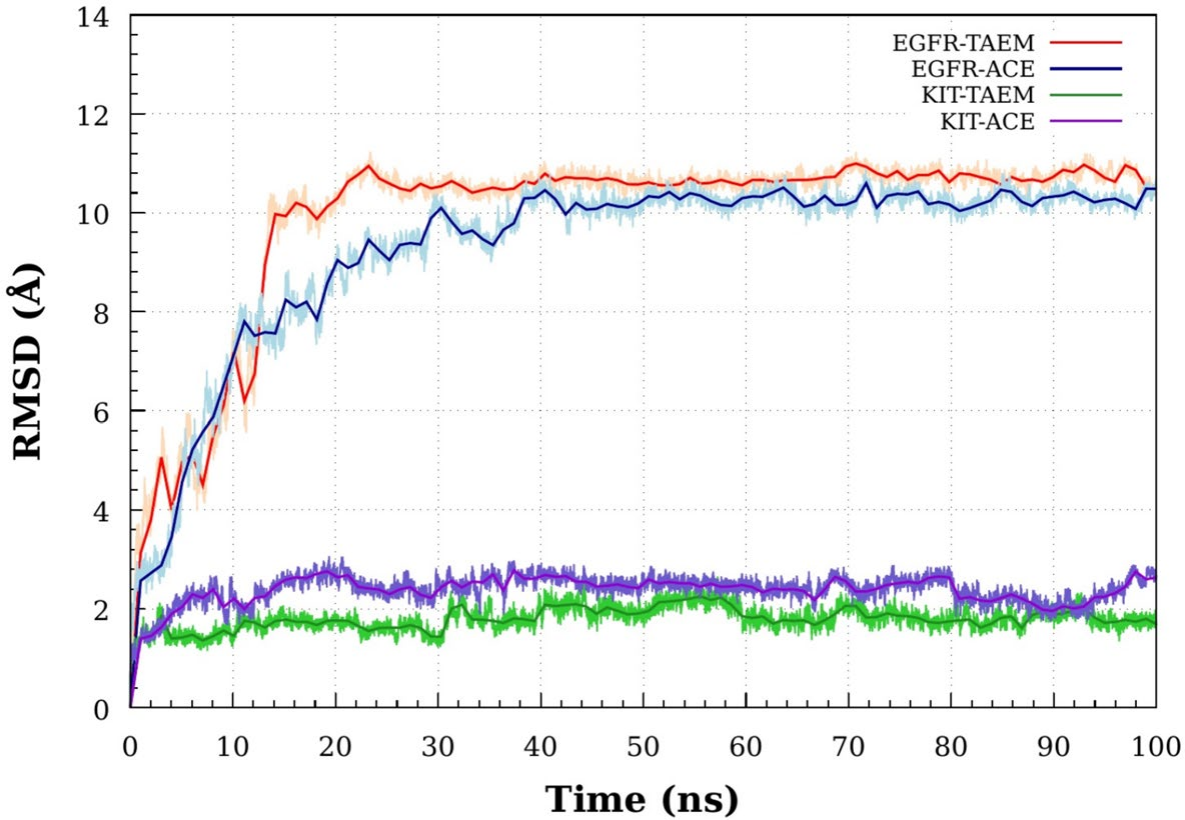
RMSD plot of *EGFR* and *KIT* without ligand

**Fig. 9:**
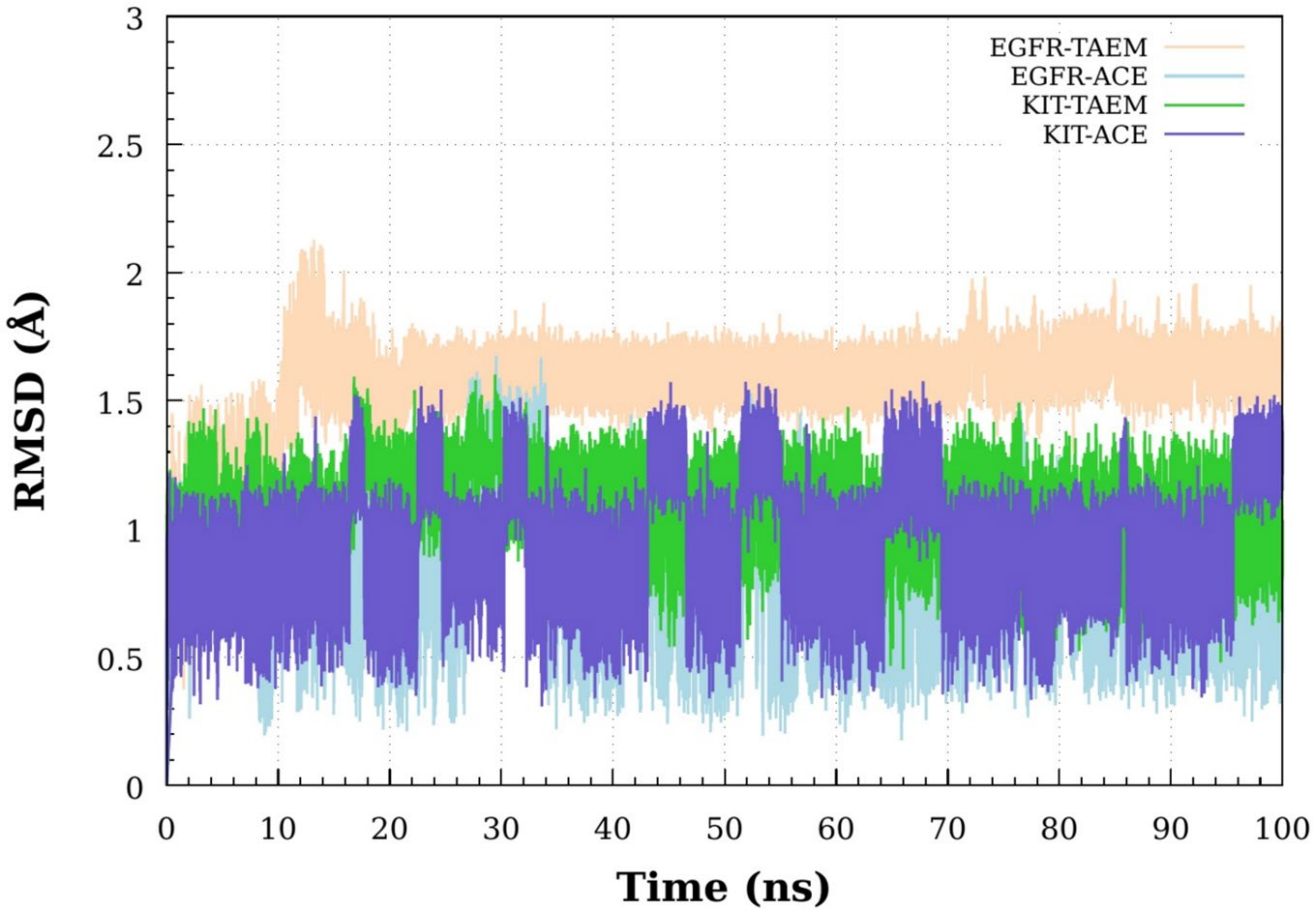
RMSD plot of two individual ligands binding to *EGFR* and *KIT*

Molecular dynamics simulations for 100 ns were performed to evaluate the structural stability and dynamics of the protein–ligand complex by analyzing the parameters RMSD, RMSF, radius of gyration (Rg), protein–ligand distance, and MM-GBSA energy. MDS was performed on two ligand complexes with each protein receptor that had the best docking scores. The RMSD plot of the protein–ligand complex is shown in Fig. 7. The conformational changes of the structure were analyzed by calculating the root mean square deviation (RMSD) of the Cα atom. Structures with RMSD values ​​ < 3 Å reflect stable conformations [33, 34]. RMSD analysis of the *EGFR*-TAEM complex and the *EGFR*-ACE complex showed significant fluctuations in the first 20 ns with average RMSD values ​​throughout the simulation of 9.98 Å and 9.30 Å, respectively. The *KIT*-TAEM complex and the *KIT*-ACE complex have low average RMSD values ​​of 2.49 Å and 2.94 Å, respectively. In addition, the RMSD graphs were also analyzed individually for the protein backbone (Fig. 8) and ligand (Fig. 9) throughout the simulation. The protein backbone atoms of the *EGFR*-TAEM and *EGFR*-ACE complexes have average RMSD values ​​of 9.92 Å and 9.30 Å, respectively, with significant fluctuations in the first 20 ns. For the proteins of the *KIT*-TAEM and *KIT*-ACE complexes, they show consistent structural stability with average RMSD values ​​of 1.80 Å and 2.38 Å, respectively. On the other hand, the average RMSD values ​​of the ligands of the *EGFR*-TAEM and *EGFR*-ACE complexes are 1.57 Å and 0.83 Å, respectively. For the *KIT* system, the lowest average RMSD value was found in *KIT*-ACE at 0.83 Å followed by *KIT*-TAEM at 1.09 Å. The low ligand RMSD values ​​in the *KIT* and *EGFR* systems indicate that the ligand is relatively stable in the binding pocket. Meanwhile, the low stability of *EGFR* indicates that the presence of the ligand does not significantly affect the protein stability.

**Fig. 10:**
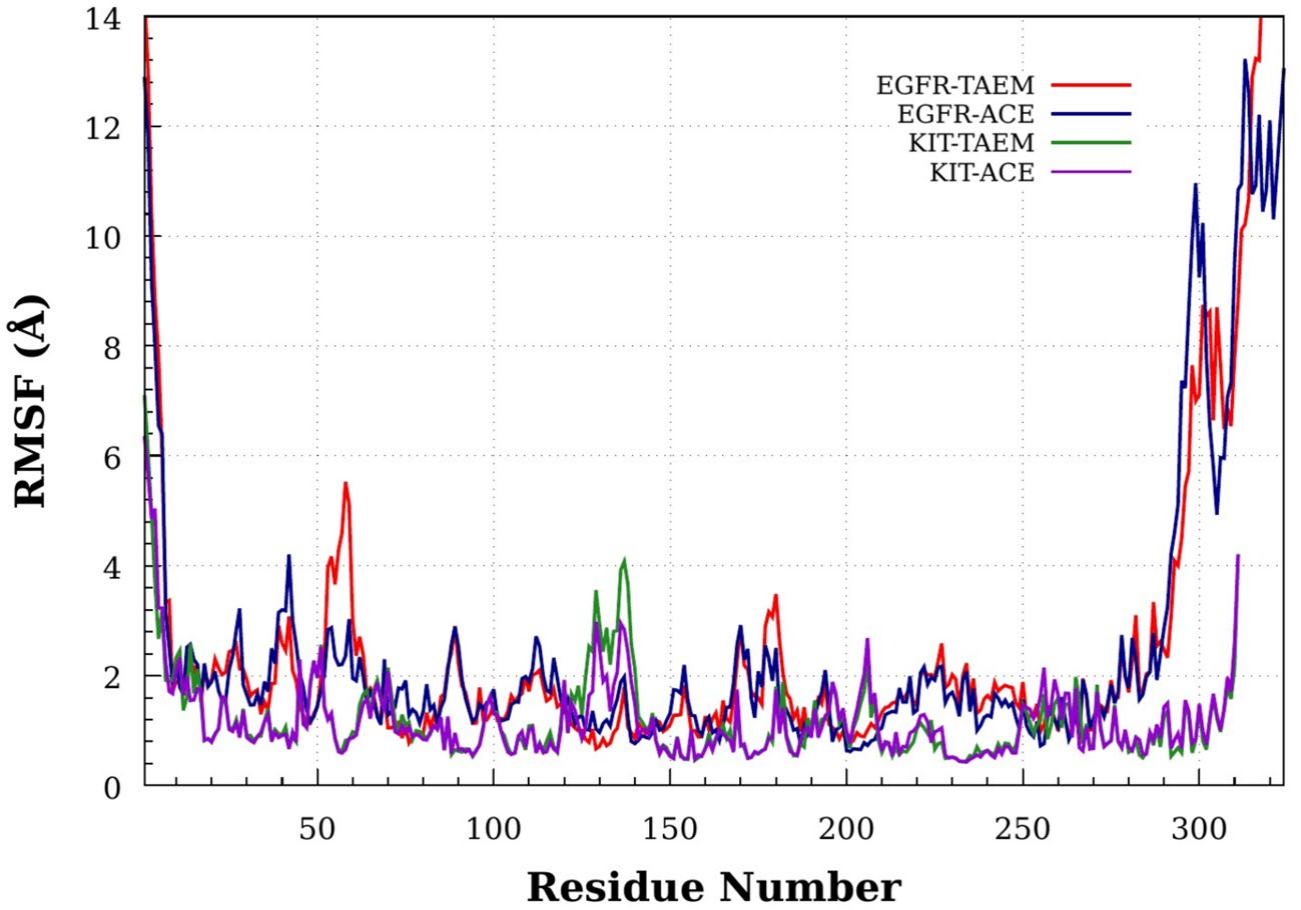
RMSF Plot of *EGFR* and *KIT* without ligand

Fluctuations in protein residues were analyzed using RMSF. In the *KIT* system, protein residues from the *KIT*-ACE and *KIT*-TAEM complexes showed average RMSF values ​​of 1.17 Å and 1.21 Å, respectively. In contrast, *EGFR* protein residues bound to TAEM and ACE ligands had higher average RMSF values ​​of 2.63 Å and 2.53 Å (Fig. 10). RMSF evaluation showed that all complexes in the *KIT* system had better conformational stability with minimum fluctuations compared to the *EGFR* system.

**Fig. 11:**
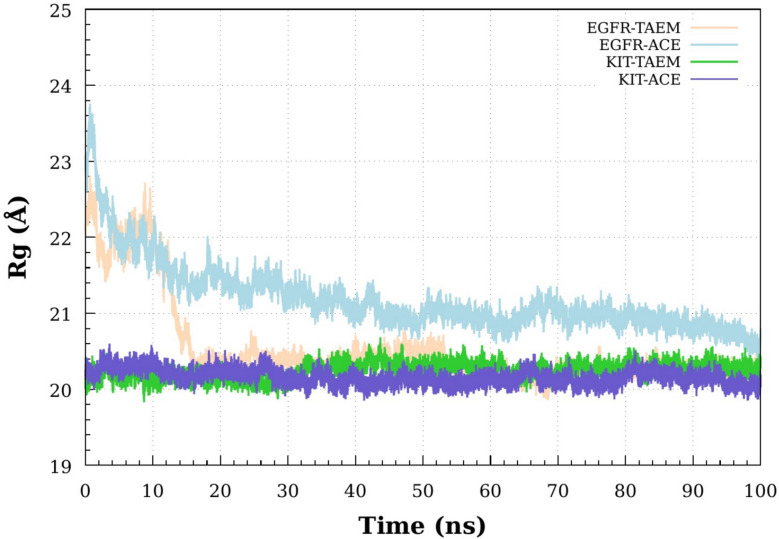
Plot of the radius of gyration (Rg) of *EGFR*-ligand and *KIT*-ligand complexes

**Fig. 12:**
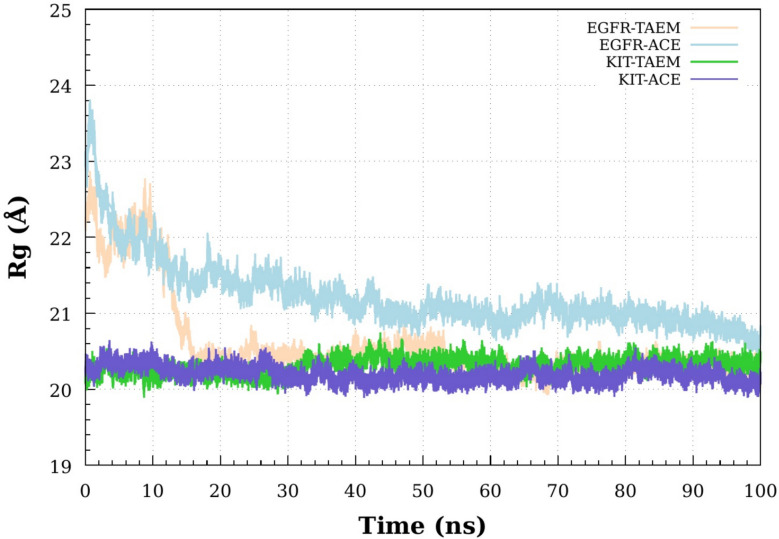
Plot of the radius of gyration (Rg) of *EGFR* and *KIT* without ligand

**Fig. 13:**
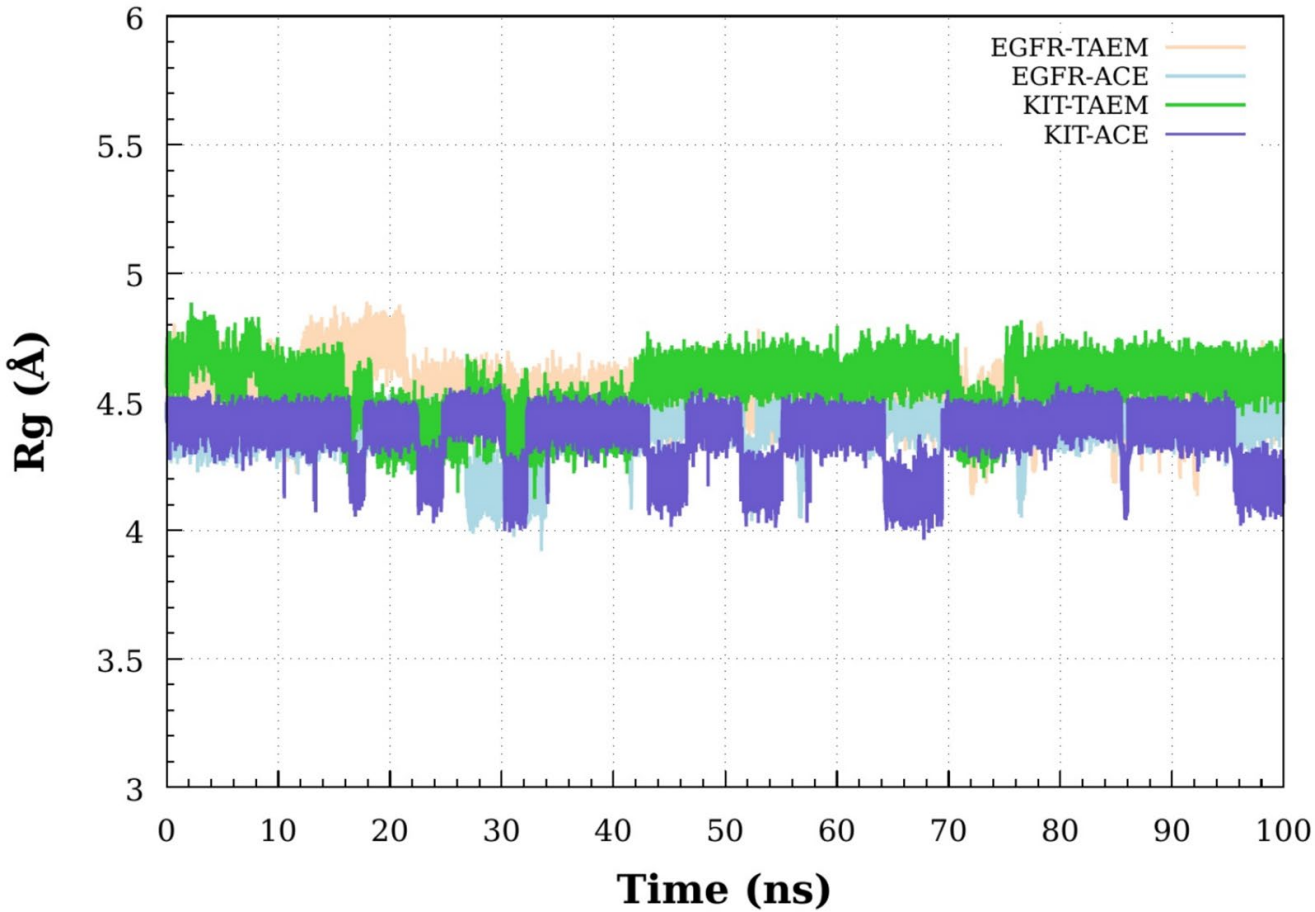
Plot of the radius of gyration (Rg) of two individual ligands binding to *EGFR* and *KIT*

The degree of protein structural density and compaction during simulation was analyzed using radius of gyration (Rg). Rg analysis was performed at three system levels, namely the whole protein–ligand complex, protein alone, and ligand alone, as shown in Figs. 11, 12 and 13. The results of the analysis showed that the average Rg values ​​for the *EGFR*-TAEM, *EGFR*-ACE, *KIT*-TAEM, and *KIT*-ACE complexes were 20.53 Å, 21.19 Å, 20.24 Å, and 20.16 Å, respectively (Fig. 11). In the *EGFR* protein (protein alone) interacting with TAEM and ACE, the average Rg values ​​were 20.59 Å and 21.23 Å, respectively. The average Rg values ​​for the *KIT* protein from the *KIT*-TAEM and *KIT*-ACE complexes were 20.30 Å and 20.20 Å, respectively (Fig. 12). Meanwhile, the average Rg value is 4.52 Å for TAEM and 4.40 Å for ACE interacting with *EGFR* protein. In the *KIT* system, the average Rg values ​​for TAEM and ACE ligands are 4.55 Å and 4.36 Å, respectively (Fig. 13). Based on Figs. 11, 12 and 13, the ligands (TAEM and ACE) and the *KIT* system maintain relatively stable Rg values ​​throughout the simulation, while *EGFR* undergoes gradual compaction. The relatively stable Rg value indicates that the structure is in a stable folded state.

**Fig. 14:**
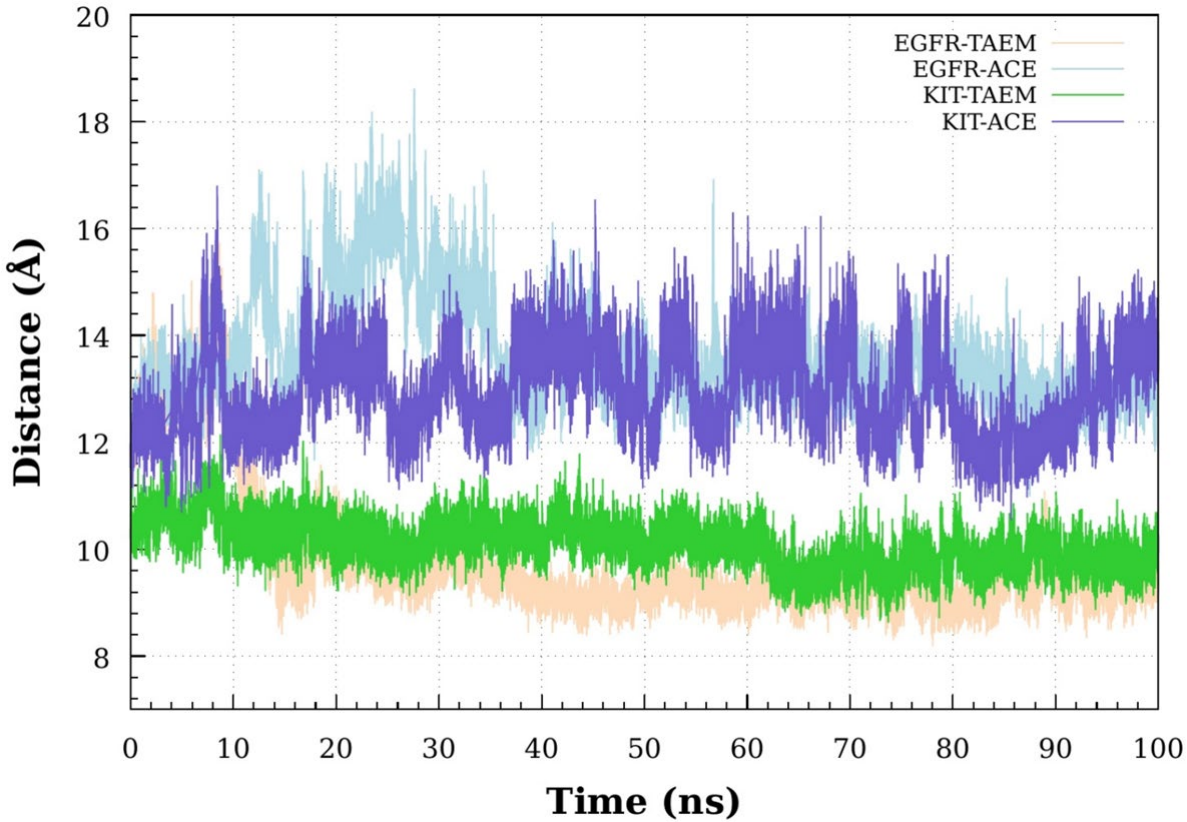
Plot of changes in protein–ligand distance in *EGFR*-ligand and *KIT*-ligand complexes

The changes in the distance between the ligand and the active residue of the target protein are shown in Fig. 14. The *KIT*-TAEM complex shows a relatively stable protein–ligand distance throughout the simulation, while the *EGFR*-TAEM complex experiences a sharp decrease in the protein–ligand distance in the first 15 ns. This result is confirmed by the low average protein–ligand distance values ​​of the *EGFR*-TAEM and *KIT*-TAEM complexes, which are 9.78 Å and 10.10 Å, respectively. Meanwhile, the *KIT* and *EGFR* proteins interacting with ACE show significant fluctuations throughout the simulation. The *KIT*-ACE and *EGFR*-ACE complexes have large average protein–ligand distance values, which are 12.96 Å and 13.61 Å, respectively.

**Fig. 15:**
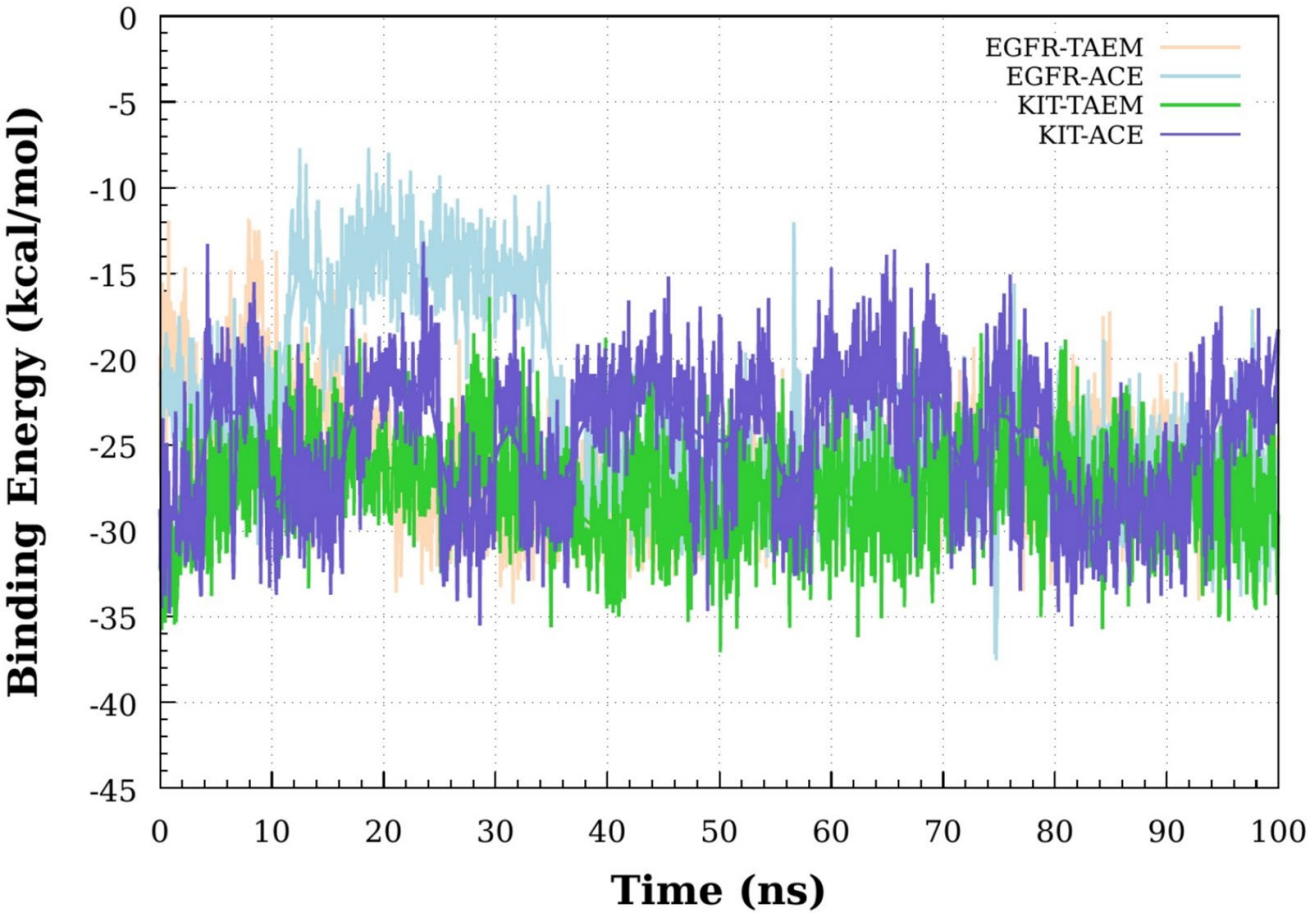
Free binding energy (in kcal/mol) of the docked complex calculated using MMGBSA analysis

The binding free energy of each complex was calculated using the MMGBSA method. As shown in the Fig. 15, the *KIT*-TAEM and *EGFR*-TAEM complexes exhibit relatively stable binding free energy profiles throughout the simulation with average ${\Delta G}_{binding}$ values ​​of −27.63 kcal/mol and −26.31 kcal/mol, respectively. For the *KIT*-ACE complex, the system fluctuates throughout the simulation with an average ${\Delta G}_{binding}$ value of −24.84 kcal/mol. Meanwhile, the *EGFR*-ACE complex experiences significant fluctuations in the first 10–35 ns time span. The average ${\Delta G}_{binding}$ value of the *EGFR*-ACE complex throughout the simulation is −23.39 kcal/mol. Based on the data in Table 2, it can be concluded that van der Waals interactions contribute greatly to the stabilization of the complex.

**Table 2:** Of Binding free energies for *EGFR*-ligand and *KIT*-ligand complexes calculated using MMGBSA analysis

Ligand	${E}_{vdw}$	${E}_{ele}$	${E}_{GB}$	${E}_{SURF}$	${\Delta G}_{gas}$	${\Delta G}_{solv}$	${\Delta G}_{binding}$
*KIT*-TAEM	−42.82	−22.46	43.19	−5.55	−65.28	37.65	−27.63
*EGFR*-TAEM	−42.42	−6.39	27.98	−5.48	−48.81	22.50	−26.31
*KIT*-ACE	−34.86	−6.06	20.42	−4.34	−40.92	16.08	−24.84
*EGFR*-ACE	−32.72	−11.51	24.98	−4.15	−44.22	20.83	−23.39

## Discussion

In this study, we employed in silico network pharmacology to predict potential targets of three emodin derivatives: emodin, ACE, and TAEM, using the Swiss Target Prediction database. Emodin was selected as the lead compound for HCC drug development due to its known cytotoxicity, yet potential for structural optimization. Structural modifications, such as acetylation, aim to enhance its pharmacodynamic properties. Emodin contains three hydroxyl groups; however, due to intramolecular hydrogen bonding between the 1,8-dihydroxy and 9-carbonyl groups, the 3-hydroxyl group remains the most reactive and suitable for derivatization under appropriate conditions.

Network pharmacology has transformed drug discovery by integrating systems biology and polypharmacology to identify multi-target interactions, enabling drug repurposing and elucidating mechanisms of action [35–37]. In our analysis, target prediction revealed significant overlap with HCC-related targets, notably *EGFR* and *KIT*, which are known to drive tumor progression and metastasis [38–43]. This approach offers a holistic, cost-effective strategy for identifying promising compounds and optimizing their therapeutic profiles [44].

Molecular docking simulations were conducted to evaluate the binding affinities of the compounds with *EGFR* and *KIT*, revealing that TAEM had the highest affinity for both targets. This was consistent with in vitro cytotoxicity results, where TAEM demonstrated superior activity against HepG2 cells. To further validate these findings, 100 ns MDS were performed on the top ligand-target pairs, analyzing RMSD, RMSF, radius of gyration (Rg), protein–ligand distance, and MM-GBSA binding energy.

MDS indicated that the *KIT*-TAEM and *KIT*-ACE complexes were more stable, with lower average RMSD values (2.49 Å and 2.94 Å, respectively), whereas *EGFR* complexes displayed higher fluctuations, particularly in the first 20 ns. RMSF analysis supported these results, showing lower residue flexibility in *KIT* complexes (1.17 Å and 1.21 Å) compared to *EGFR* (2.63 Å and 2.53 Å). Rg analysis showed compact and stable conformations in *KIT* complexes, while *EGFR* systems showed gradual compaction. Protein–ligand distance analysis confirmed stable binding in *KIT*-TAEM, whereas *EGFR*-TAEM experienced early-phase instability. MM-GBSA results revealed that *KIT*-TAEM (−27.63 kcal/mol) and *EGFR*-TAEM (−26.31 kcal/mol) had the most favorable binding energies, with van der Waals forces as key contributors. In contrast, ACE-containing complexes, especially *EGFR*-ACE, showed less stable binding and higher energy fluctuations (−23.39 kcal/mol).

*EGFR* and *KIT* are crucial in the progression and metastasis of HCC [39–43]. *EGFR* functions as a major signaling hub for inflammatory mediators and interacts with key pathways. It has been shown to induce *CXCL8* and *CXCL5* production in a dose- and time-dependent manner, which can be attenuated by *EGFR* inhibition, highlighting its therapeutic relevance [40, 45]. *KIT* is involved in stem cell activation and liver regeneration, with *KIT*-positive HCC patients exhibiting improved survival outcomes, suggesting its prognostic and therapeutic significance [42, 43, 46].

Nonetheless, this study has limitations. First, although in silico methods are efficient for target identification, they remain predictive and require experimental validation. Second, while TAEM demonstrated promising cytotoxicity in vitro, no in vivo studies or pharmacokinetic assessments have been conducted. Third, this study focused only on *EGFR* and *KIT* among the predicted targets, limiting the broader applicability of the results. Lastly, the limited number of predicted targets for TAEM (only one) may stem from constraints in the prediction algorithm rather than its actual biological scope. Despite these limitations, our findings illustrate how combining network pharmacology, docking, and MDS can aid in prioritizing candidate compounds. Among them, TAEM emerges as a strong candidate for further investigation in biological models to validate its potential as a therapeutic agent for HCC.

## Conclusion

This study applied integrated in silico methods to investigate emodin derivatives as candidate therapeutics for HCC. TAEM emerged as a promising compound, showing high binding affinity to *EGFR* and *KIT*, favorable molecular dynamics stability, and greater in vitro cytotoxicity compared to emodin and ACE. Among the compounds tested, the *KIT*–TAEM complex showed the most consistent stability and binding energy profile. While the findings are encouraging, they remain preliminary. Additional work is needed to confirm the pharmacokinetic properties, safety profile, and therapeutic efficacy of TAEM in vivo. Nonetheless, the combined approach used here illustrates the potential of computational screening for identifying and optimizing anti-cancer candidates from natural products.

## Supplementary Information

Supplementary Material 1. Supplementary Table 1. Summary of 3494 HCC-associated genes from cBioPortal

Supplementary Material 2. Supplementary Table 2.Lists of compound targets gathered from the Swiss Target Prediction

Supplementary Material 3. Supplementary Table 3. Summary of overlapped 20 target genes between emodin derivates and HCC

Supplementary Material 4. Supplementary Table 4.Functional enrichment analysis

## Data Availability

Data is provided within the supplementary information files.
